# Liquid-Solid Phase-Inversion PLGA Implant for the Treatment of Residual Tumor Tissue after HIFU Ablation

**DOI:** 10.1371/journal.pone.0117358

**Published:** 2015-02-24

**Authors:** Juan Li, Tianyi Krupka, Jinpeng Yao, Ronghui Wang, Lin Jiang, Yang Zhou, Guoqing Zuo, Zhibiao Wang, Lili Dai, Jianli Ren, Yuanyi Zheng, Dong Wang

**Affiliations:** 1 Second Affiliated Hospital and Ultrasound Imaging Institute, Chongqing Medical University, Chongqing, 400010, P. R. China; 2 Radiology Department, Stanford University, Stanford, California, United States of America; 3 College of Biomedical Engineering, Chongqing Medical University, Chongqing, 400016, P. R. China; 4 Department of Ultrasound, Children’s Hospital of Chongqing Medical University, Ministry of Education Key Laboratory of Child Development and Disorders, Chongqing, 400014, P. R. China; Indian Institute of Technology, INDIA

## Abstract

**Background:**

HIFU has been shown to be a more suitable alternative for the treatment of primary solid tumors and metastatic diseases than other focal heat ablation techniques due to its noninvasive and extracorporeal nature. However, similar to other focal heat ablation techniques, HIFU is still in need of refinements due to tumor recurrence.

**Methods:**

In this work, we investigated the effectiveness of an adjunct treatment regimen using doxorubicin (DOX)-loaded, injectable, in situ-forming, and phase-inverting PLGA as the second line of defense after HIFU ablation to destroy detrimental residual tumors and to prevent tumor recurrence. All of the statistical analyses were performed using the Statistical Package for the Social Sciences 18.0(SPSS, Inc., Chicago, IL, USA), and *p*< 0.05 was considered statistically significant. All of the results are presented as the means ± STDEV (standard deviation). For multiple comparisons, ANOVA (differences in tumor volumes, growth rates, apoptosis, proliferation indexes, and Bcl-2 and Bax protein levels) was used when the data were normally distributed with homogenous variance, and rank sum tests were used otherwise. Once significant differences were detected, Student-*t* tests were used for comparisons between two groups.

**Results:**

Our results revealed that DOX diffused beyond the ablated tissue regions and entered tumor cells that were not affected by the HIFU ablation. Our results also show that HIFU in concert with DOX-loaded PLGA led to a significantly higher rate of tumor cell apoptosis and a lower rate of tumor cell proliferation in the areas beyond the HIFU-ablated tissues and consequently caused significant tumor volume shrinkage (tumor volumes:0.26±0.1,1.09±0.76, and 1.42±0.9cm^3^ for treatment, sham, and no treatment control, respectively).

**Conclusions:**

From these results, we concluded that the intralesional injection of DOX-loaded PLGA after HIFU ablation is significantly more effective than HIFU alone for the treatment of solid tumors.

## Background

High-intensity focused ultrasound (HIFU) has been receiving increased attention for the treatment of oncology. Compared with diagnostic ultrasound (intensities: 0.1–100mW/cm^2^), HIFU (intensities: 100–10,000W/cm^2^) uses significantly higher time-averaged intensities in the focal zone of the ultrasound transducer. With such high acoustic intensities, the tissue in the path of the ultrasound beam absorbs the acoustic energy, and this absorption generates heat and causes time and temperature-dependent coagulation necrosis of tissues [1–3], therefore, HIFU is considered a viable, cost-effective and noninvasive focal heat ablation method for the treatment of solid tumors. The therapy is localized and thus does not cause systemic toxicity. Even more attractive, HIFU provides the option of preserving certain organs, such as the uterus and breasts, which are often severed in open surgery protocols. Despite these advantages, this technology still bears a number of drawbacks similar to those of other focal heat ablation techniques (radiofrequency, microwave, and laser), such as local tumor recurrence caused by insufficient heating of the tumor tissue [4–6], and hence needs refinement.

As adjuncts to focal heat ablation techniques, polymer drug delivery systems have long been explored. Among them, PLGA polymers have been generating great interest due to their excellent bio-compatibility, biodegradability, and mechanical strengths [7, 8]. More important, PLGA polymers have been approved by the FDA in the United States for drug delivery purposes. Pre-manufactured PLGA implants in the form of millirods and other forms have been extensively studied. The results from these studies have shown that PLGA millirods are compatible with a biological environment, effective for the release of DOX and other drugs in a sustained and controlled manner [9–11], and effective for enhancing tumor treatment after radiofrequency ablation.

Recently, an injectable version of these pre-formed PLGA implants were also investigated in vitro and in vivo in subcutaneous tumors of rats. The results from these studies demonstrated that the in situ formation of PLGA drug delivery systems is a more attractive alternative due to various advantages, such as simple formulation processes, less invasive placements, and in situ formation natures. Although extensive efforts have been placed on drug release and implants and the in situ formation characteristics of these drug delivery systems, most of these works were performed with model drugs, such as fluorescein, and few studies have been performed to the show the in vivo efficacy of these systems in the enhancement of focal heat ablation.

The purpose of this work was to devise and test an adjunct treatment regime for solid tumors using the combination of HIFU, PLGA, and DOX. In this regime, HIFU provided the primary means to destroy cancer tissue at the target site. DOX, a real cancer drug, provided the second line of defense and destroyed all potential residual tumor cells. The easily formulated injectable in situ-forming PLGA gel was injected into the ablated tissue after HIFU and served as a depot for the controlled and localized release of DOX.

To the best of our knowledge, this study provides the first demonstration of the combination of HIFU and an injectable in situ-forming PLGA implant for cancer treatment and provides a large quantity of in vivo efficacy data.

## Materials and Methods

### 1. Materials

All of the materials were used as received with no further purification. Poly (D, L-lactide-co-glycolide) (PLGA 50:50, Mw20,000) was obtained from Daigang, China. Doxorubicin hydrochloride (DOX, Mw579.98) was purchased from Chongqing Medicine Co. (Chongqing, Beijing, China). Hanks’ balanced salt solution was obtained from Solarbio (Beijing, China). 1X PBS was obtained from Maizin (Fuzhou, China). Agarose was obtained from Biowest (Wuhan, China). Hematoxylin and eosin (H&E), PCNA immunohistochemistry (IHC) chemicals, and glycerol were obtained from Boster (Wuhan, China). The TUNEL assay kit was obtained from Roche (Switzerland). Pentobarbital was obtained from Shanghai Chemical Reagent Factory (Shanghai, China). Na_2_S was obtained from Sinpeuo (Shanghai, China). Streptomycin was obtained from NCPC (Shijiazhuang, China). Microbubble contrast agents were made in our laboratory. The lipids 1, 2-dipalmitoyl-sn-glycero-3-phosphocholine (DPPC, Mw: 734.05), 1, 2-dipalmitoyl-sn-glycero-3-phosphate (DPPA, Mw:670.88), 1, 2-distearoyl-sn-slycero-3-phosphoethanolamine(DSPE, Mw:748.07), 1-methyl-2-pyrrolidone (NMP) were obtained from Sigma Aldrich (St. Louis, MO, USA). Octafluoropropane (C3F_8_) was obtained from Tianjin Summit Specialty Gases Ltd. (Tianjin, China).

### 2. Formulation of microbubble contrast agents

The microbubble ultrasound contrast agents were prepared. We dissolved 5mg of DPPC, 1mg of DPPA, 2mg of DSPE, and 50μL of glycerol in 1ml of 1X PBS in an incubator for 30 min at 40°C. The vials were then sealed, and air was withdrawn. C3F_8_ was added to the vials until the pressure in the vial was equalized. The vials were then mechanically agitated for 45s in a dental amalgamator (YJT, Shanghai Medical Instruments Co., Ltd.).

### 3. Preparation of doxorubicin (DOX) gel

The contents of the doxorubicin (DOX) gel were DOX hydrochloride, poly, and NMP. First, the PLGA polymer was added to the NMP solution in scintillation vials. The scintillation vials were then placed at 37°C in an orbital shaker. After the PLGA was completely dissolved, which took a few hours, DOX hydrochloride was added to the dissolved PLGA solution. The homogenous solutions were then stored at 4°C until further use. To establish this work, three different formulations of NMP:PLGA:doxorubicin hydrochloride at mass ratios of 80 (or 60 or 40)/15 (or 35 or 55)/5 were prepared and preliminarily tested.

### 4. In vitro drug release and degradation

The drug release profile from the formulation with a NMP/PLGA/DOX hydrochloride mass ratio of 40/55/5 was examined in vitro. Briefly, 200μL of DOX gel (n = 3) was injected into a dialysis membrane bag (Mw: 500–1000) that contained 2ml of 1X PBS. The dialysis membrane bags were individually placed in flasks containing a bath solution of 100ml of 1X PBS (pH 7.4) and placed in an orbital incubator-shaker set at 37°C and 60rpm (revolutions per minute). Five milliliters of the bath solutions were then sampled at 1, 2, 4, 8, and 12h and then every 24h up to 7 days. After each sampling, 5ml of fresh 1X PBS was supplemented. The concentrations of the released DOX were quantified by high-performance liquid chromatography (HPLC, Agilent 1100, USA) at its maximum adsorption wavelength (λ = 254nm). The cumulative drug releases at each sampling time were calculated by normalizing to the total injected drug mass, which was determined by the degradation of the implant at the end point of the study.

The DOX degradation in 1X PBS was also monitored concurrently during the drug release experiments. Briefly, DOX was dissolved in 1X PBS. At each sampling time as in the drug release experiments (1, 2, 4, 8, and 12h and then every 24h up to 7 days), 1ml of the drug solution was sampled, and the concentration of doxorubicin in each sample was quantified using HPLC, at its maximum adsorption wavelength (λ = 254nm).

### 5. Implantation of VX_2_ subcutaneous tumors

All of the animal experiments were conducted following a protocol approved by the animal ethics committee of Chongqing Medical University. A tumor-bearing rabbit with a VX_2_ tumor in the thigh was obtained from the laboratory of Ultrasound Engineering Institute of Chongqing Medical University. New Zealand White rabbits (2.0–2.5kg at ages between 2 and 3 months), which were used as recipient animals, were purchased from and bred in the Animal Center of Chongqing Medical University under standard conditions according to the Institute’s animal care guidelines.

A subcutaneous tumor model was developed according to a previously reported method. Under sterile and anaesthetic conditions, tumor tissue with a fish-meat-like appearance was excised from the tumor-bearing rabbit, soaked in 20ml of Hanks’ balanced salt solution, and then sheared to small pieces with a size of approximately 0.5–1.0mm in diameter. The final suspension was extracted into a 20ml syringe, and 1ml of the tumor tissue suspension was injected into the mammary gland of recipient rabbits underneath the left second nipple. Five hundred thousand units of streptomycin were administered to each animal after tumor implantation. In this work, 48 rabbits in total were used, and all of the rabbits received tumor transplants.

### 6. In vitro implant formation ultrasound imaging

The in vitro phase-inverting process of the implants was imaged using a Philips-iU22 diagnostic ultrasonic instrument (Philips Medical Systems, Bothell, WA, USA) in a custom tissue phantom with a 2cm-diamter bottom-sealed cylindrical well. The custom-made tissue phantom was fabricated similarly as in a previously published work [12, 13]. The phantoms were composed of 1% agarose. A 5–12MHz transducer was used at a mechanical index (MI) of 0.6, gain of 69%, and dynamic range of 40dB. The transducer, which was secured by a clamp, was placed in an upward position, and the tissue phantom held by a custom-made holder was positioned directly above the transducer during the imaging process. After a drop of 100μL of PLGA was placed in the phantom wells, which were prefilled with degassed water, the centers of the phase-inverting implants were located by adjusting the transducer positions. Five images of these phase-inverting implants were acquired at 1, 10, and 30s, 1, 10, and 30min, 1, 2, 4, 8, and 12h, and 1, 3, 5, and 7 days. All of the images were acquired at the same plane of view throughout the study. The average grayscale values of these images at each time point were analyzed using a Sonomath quantitative ultrasound image analyzer (Anbijie Ltd., Chongqing, China).

### 7. In vivo implant formation ultrasound imaging in ablated subcutaneous VX_2_ tumors

Three weeks after tumor transplantation, the tumor-bearing rabbits (n = 3) were anesthetized through an intramuscular injection of 3% pentobarbital solution (1ml/kg). The second breasts, where the tumors were transplanted, were carefully depilated with 8% Na_2_S. The rabbits were restrained on the treatment bed in the prone position. The depilated area was completely immersed in degassed water. The tumor was located and measured with integrated diagnostic US. 100μL of PLGA solution was slowly injected into the tumor using an 18-gauge lumbar puncture needle. Images of the phase inverting implants were acquired using a Philips IU22 diagnostic ultrasound with a 5–12MHz transducer (MI = 0.6, gain = 69%, dynamic range = 40 dB). The images from the in vivo studies were acquired at 1s, 30min, 2, 4, and 8h, and 1, 3, 5, and 7 days post injection. As in the in vitro study, five images were acquired at each time point and each interface for each implant. The average grayscale values of these images were analyzed semi-quantitatively using a Sonomath quantitative ultrasound image analyzer.

### 8. HIFU ablation

HIFU ablation was performed using a JC200 HIFU tumor ablation instrument (Chongqing Haifu Technology, Chongqing, China). This instrument consists of both a therapeutic and a diagnostic ultrasound unit under the control of a central processing system. The therapeutic transducer has a focal length of 145mm, a diameter of 220mm, and an operating frequency of 0.94MHz. The focused US transducer emits high-intensity ultrasound to target and destroy the tissue of interest. The diagnostic transducer is located in the center of the therapeutic transducer to guide and monitor the therapeutic procedures in real time. Degassed water was used as the medium.

The HIFU ablations were performed on VX_2_ tumors three weeks after transplantation. The tumor was located and measured with integrated diagnostic US before ablation. 42 rabbits were randomly divided into three groups: No Treatment (**group A**, n = 12), HIFU combined with drug-free PLGA, i.e., Sham (**group B**, n = 14), and HIFU combined with doxorubicin carrying PLGA, i.e., Treatment (**group C**, n = 16). However, to artificially create residual tumors, approximately two thirds of the total tumor volume of each tumor was ablated. The HIFU ablation parameters were maintained constant as mentioned above with an acoustic power of 250W and an exposure duration of 5s.

### 9. Treatments of residual VX_2_ tumors using DOX-loaded PLGA gel

Following HIFU, the post-ablation tumors of groups **B** and **C** were also given percutaneous injections of 0.1ml of DOX-free PLGA and DOX-containing PLGA, respectively. The length and depth of the tumors were measured using ultrasound before and after treatment. The volume of the tumor was calculated by the following equation: V = L×D^2^ /2, where L is the length and D is the depth. Half of the animals in each group were killed with a pentobarbital solution overdose 4 and 8 days after treatment. After tumor removal, each tumor was dissected into two relatively equal sections and photographed. The tumor tissues were prepared for further analysis.

### 10. In vivo contrast-enhanced ultrasound tumor imaging with and without HIFU ablation

Six additional New Zealand white rabbits carrying subcutaneous VX_2_ tumors were divided into two groups. B-mode ultrasound with a 5–12MHz transducer (MI = 0.6, gain = 69%, dynamic range = 40 dB) without contrast enhancements was used for all of the tumors. Then, three rabbits intentionally received incomplete (approximately 2/3 of the total tumor volume) HIFU ablation (operating frequency:0.94MHz, power:250W, duration:5s, repetition:3.82). All of the rabbits underwent contrast-enhanced ultrasound imaging. Briefly, after the rabbits were anesthetized, 1ml of the microbubble contrast agents diluted in 5ml of 0.9% saline was administered intravenously along the edge of the ears, and this administration was followed by the administration of 1ml of 0.9% saline flush. Both ultrasound movies and images were acquired with a 5–12MHz transducer (Contrast mode).

**TTC stain**. Immediately after HIFU ablation, all of the rabbits were killed, and the entire tumors were surgically removed and sectioned into approximately 2mm-thick slices along the largest surface areas. The maximum section of necrotic tumor tissue was selected for staining at 37°C in 2% 2,3,5-triphenyltethrazolium chloride (TTC) for 15min to stain tissues with functional mitochondria.

### 11. Fluorescent imaging of DOX distribution

Eight days after receiving incomplete HIFU ablation and subsequent PLGA injection, 3/19 (n = 3) rabbits in **Group C** (incomplete HIFU ablation followed by a DOX-containing PLGA injection) were killed with an overdose of pentobarbital solution. The tumors were carefully removed surgically. The largest diameters of the tumors were identified. Along these largest diameters, the tumors were dissected into two relatively equal sections. One half of the tumor was embedded in OCT, frozen, sliced into 100μm-thick sections, and mounted on cover slips. Fluorescent images were then acquired with fluorescent microscopes at a wavelength of 512nm. The fluorescent signals were then quantified with grayscale analysis using a Sonomath quantitative image analyzer (Anbijie Ltd, Chongqing, China) at distances, r, of 0.2, 0.4, 0.6, and 0.8cm from the center of the implants. The fluorescence intensities were plotted against the distance, r, and the DOX distribution in the HIFU-ablated tumors was obtained (n = 3).

### 12. Histological Analysis and Protein Assays

Eight days after the treatments, the remaining 18 rabbits (n = 6 per group) were killed by an overdose of pentobarbital solution. The entire tumors were then carefully excised, and the tissues were divided into groups for further processing.

**12.1. Hematoxylin and eosin (H&E)**. First, a portion of the tumor tissues were fixed in 4% buffered formalin solution for at least 24h. These tissues were then processed by the Histology Core Facility in our Institution. Briefly, the tissues underwent dehydration and were embedded in paraffin. Then, microscopic sections were cut (5μm) and stained with hematoxylin and eosin (H&E). Images of the stained tissues were obtained with a light microscope. All of the specimens were inspected by two independent and trained observers.

**12.2. PCNA proliferation and TUNEL apoptosis assay**. The proliferation index was assessed through a quantitative morphometric analysis of the proliferating cell nuclear antigen (PCNA) expression, one of the established markers for cell proliferation, as described by Asai et al.[14] For PCNA localization, sections fixed in 4% formalin and embedded in paraffin were incubated for 60 min with a mouse monoclonal anti-PCNA. A peroxidase-conjugated antibody to mouse IgG (Abcam Inc., Cambridge, MA, USA) and then diaminobenzidine (Sigma Aldrich, St. Louis, Missouri, USA) was applied to localize PCNA in the tissue sections.

The DNA fragmentation was assessed by in situ terminal transferase-mediated fluorescein deoxy-UTP nick end labeling (TUNEL) using the Apoptag Peroxidase In situ Apoptosis Detection Kit (Serologicals Corp., Norcross, GA, USA).

PCNA proliferation and DNA fragmentation analysis were determined with an image analysis system by inspecting five randomly selected high-power fields (imaging magnification was 400X) outside of the necrotic region of the tumor tissue. The cells that were stained brownish yellow, chocolate, or dark brown were defined as positive for proliferation or apoptosis. In each region of 1000 cancer cells, the numbers of positive cells were defined as the proliferation index (PI) or apoptosis index (AI).

**12.3. Western blot for Bcl-2 and Bax protein levels**. The expression of Bcl-2 and Bax proteins in VX_2_ tumor tissues was determined by Western blot analysis as described previously [15]. Briefly, 30μg of proteins from each tumor tissue sample were subjected to electrophoresis on 10% sodium dodecyl sulfate (SDS)-polyacrylamide gels and then transferred to nitrocellulose membrane filters (Millipore, USA). The membrane filters were blocked in Tris-buffered saline (TBS, pH 7.5) with 0.5% Tween-20 (TBST) and 5% nonfat milk for 1 h and subsequently subjected to incubation with primary mouse anti-rabbit antibodies Bcl-2 and Bax monoclonal antibodies (Abcam, USA) at a dilution of 1:200 for 2h. Finally, the membrane filters were incubated for 30min with horseradish peroxidase-conjugated secondary goat anti-mouse (Zhongshan, Beijing, China), and anti-mouse IgG antibodies (1:4000 dilution, Western Biotechnology, China). The specific proteins were detected with the use of an enhanced chemiluminescence (ECL, Biotechnology, China) detection system (Amersham, USA). A monoclonal antibody for GADPH (1:3000 dilution, Western Biotechnology, China) was used as the control.

### 13. Statistical analysis

All of the statistical analyses were performed using the Statistical Package for the Social Sciences 18.0 (SPSS, Inc., Chicago, IL, USA), and *p* < 0.05 was considered statistically significant. All of the results are presented as the means ± STDEV (standard deviation). For multiple comparisons, ANOVA (differences in tumor volumes, growth rates, apoptosis, proliferation indexes, and Bcl-2 and Bax protein levels) was used when the data were normally distributed with homogenous variance, and rank sum tests were used otherwise. Once significant differences were detected, Student-*t* tests were used for comparisons between two groups.

## Results

### 1. In vitro drug release and degradation

**Fig 1:**
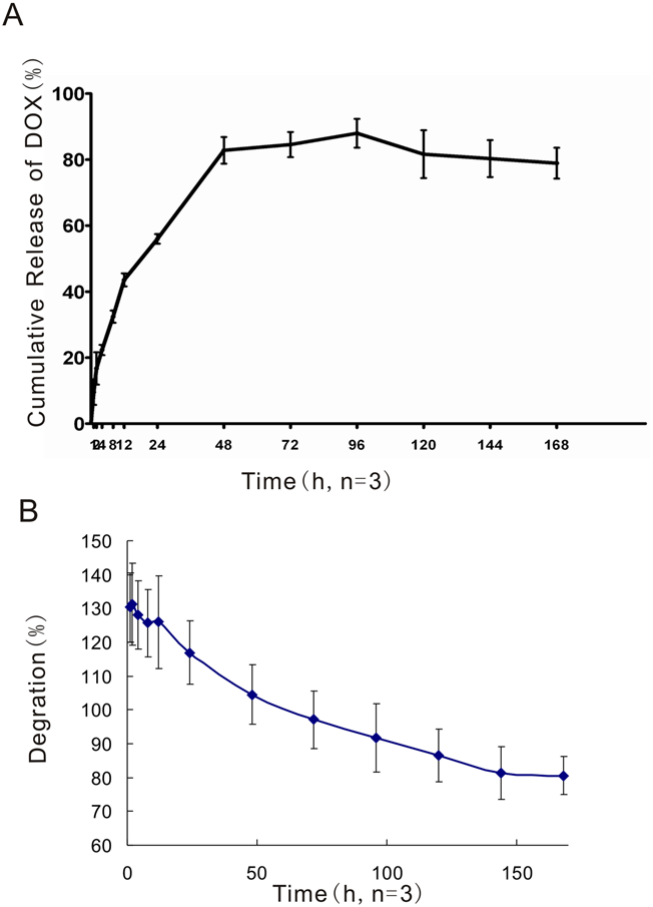
In vitro Cumulative DOX Release (%). The data are presented as the means ± STDEV (the standard deviation, n = 3).

The release of DOX from the in situ phase-inverting PLGA implants was examined in vitro. Fig. 1 shows that a total of 13.50±0.8mg of doxorubicin was released over the 7-day experimental period, which corresponds to 78.92±4.7% of the drug loaded. A burst release occurred during the first hour that resulted in the release of 9.55±3.9% of the loaded DOX. The accumulative drug release reached its maximum of 88.90±4.3% by the 4th day and then began to decrease accompanied by the degradation of the DOX gel.

### 2. In vitro implant formation (or phase inversion)

**Fig 2:**
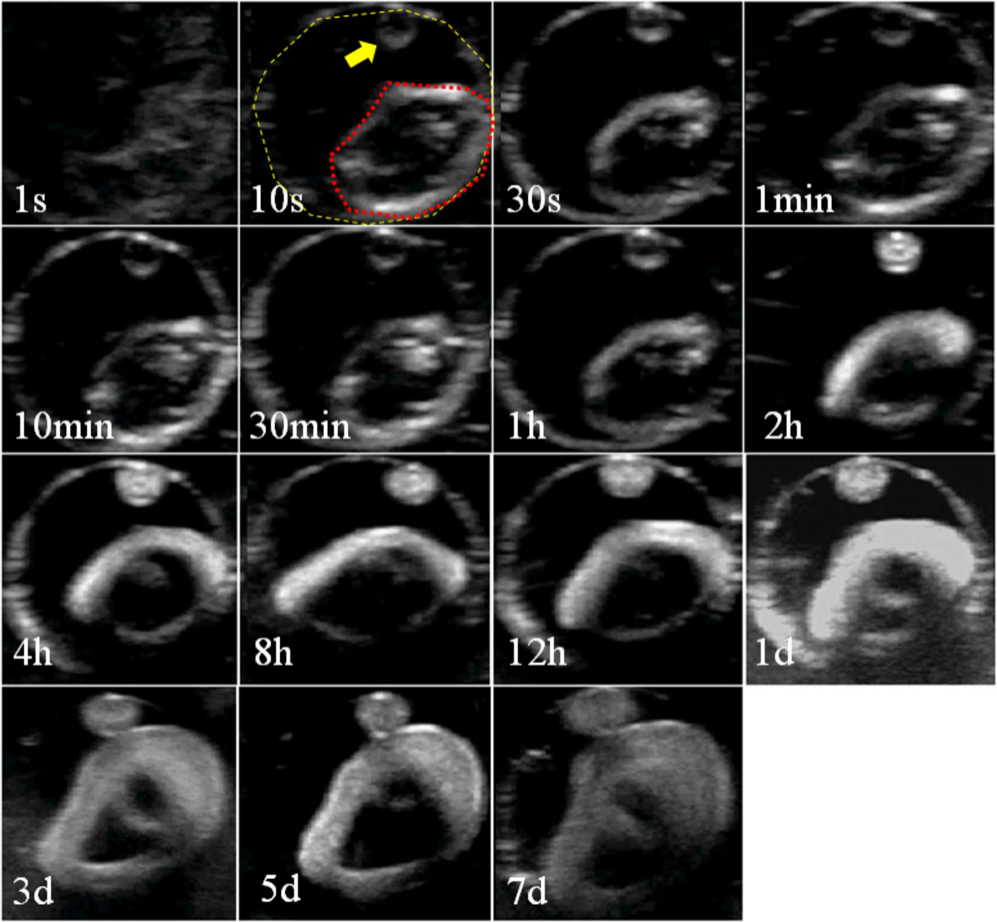
In vitro Phase Inversion Imaging. Representative grayscale ultrasound images of the phase-inversion process of the DOX-loaded PLGA gel over time in vitro. The outer circle of the dashed yellow line indicates the boundary of the well formed by the agarose phantom. The inner circle of the red dashed line indicates the PLGA gel material, and the arrow points to an unintentional small drop of PLGA gel material in the well.

NMP:PLGA:DOX at a mass ratio of 40:55:5 was studied. Three noteworthy phenomena were observed. First, the specific formulation of NMP/PLGA/DOX did undergo phase inversion in vitro. Second, the PLGA gel appeared to be hypoechoic before phase inversion and phase inverted over time to a more solid state from the outside inward, as demonstrated by a more hyperechoic donut ring on the outside of the implant and a more hypoechoic center. Finally, the PLGA gel increased in volume, i.e., swelled, over time, as indicated by the spatial relationships of the PLGA gel to the boundaries of the phantom wells (Fig. 2).

### 3. In vivo HIFU effect assessment

**Fig 3:**
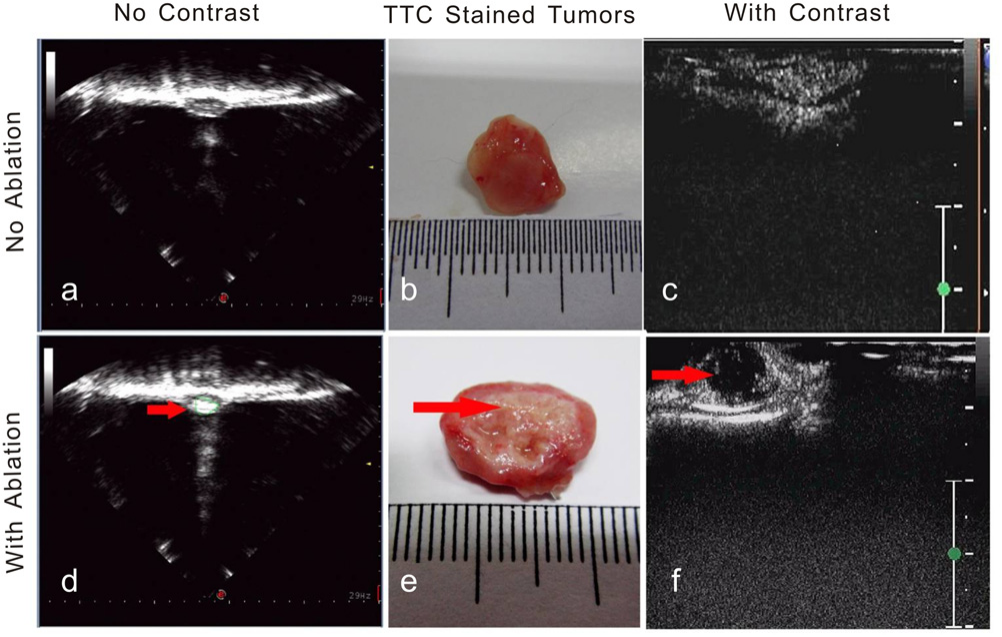
Contrast-Enhanced HIFU Ablation Imaging. Representative ultrasound tumor images with and without intentionally incomplete HIFU ablation. A: Without HIFU ablation and without contrast enhancement. B: TTC-stained tumor that was not subjected to HIFU ablation. C: Contrast-enhanced ultrasound image of tumor without HIFU ablation. D: HIFU-ablated tumor without contrast enhancement. E: TTC-stained tumor that received HIFU ablation. F: Contrast-enhanced ultrasound image of tumor with HIFU ablation. The arrows indicate the ablated area. The scale bars represent 1cm.

Fig. 3 shows representative images. Our results show that the VX_2_ tumors without HIFU ablation and without contrast enhancement are uniformly clump-like and hypoechoic. These tumors appeared to be elliptical in shape with clear tumor and normal tissue boundaries (Fig. 3A). However, HIFU ablation increased the tissue echo, and the initially hypoechoic tissue became hyperechoic (Fig. 3D). In addition, our results indicate that, without HIFU ablation, the microbubble contrast agents uniformly penetrated the entire tumor volume and enhanced the tissue contrast within 5–10s after administration (Fig. 3C). These tumors appeared rose red in color after TC staining (Fig. 3B). In contrast, after incomplete ablation, the contrast enhancement in the residual tumors tissues was irregular (Fig. 3F) and absence in the HIFU-ablated region. TTC failed to stain the HIFU-ablated tissue region (Fig. 3E).

### 4. In vivo implant formation (or phase inversion)

**Fig 4:**
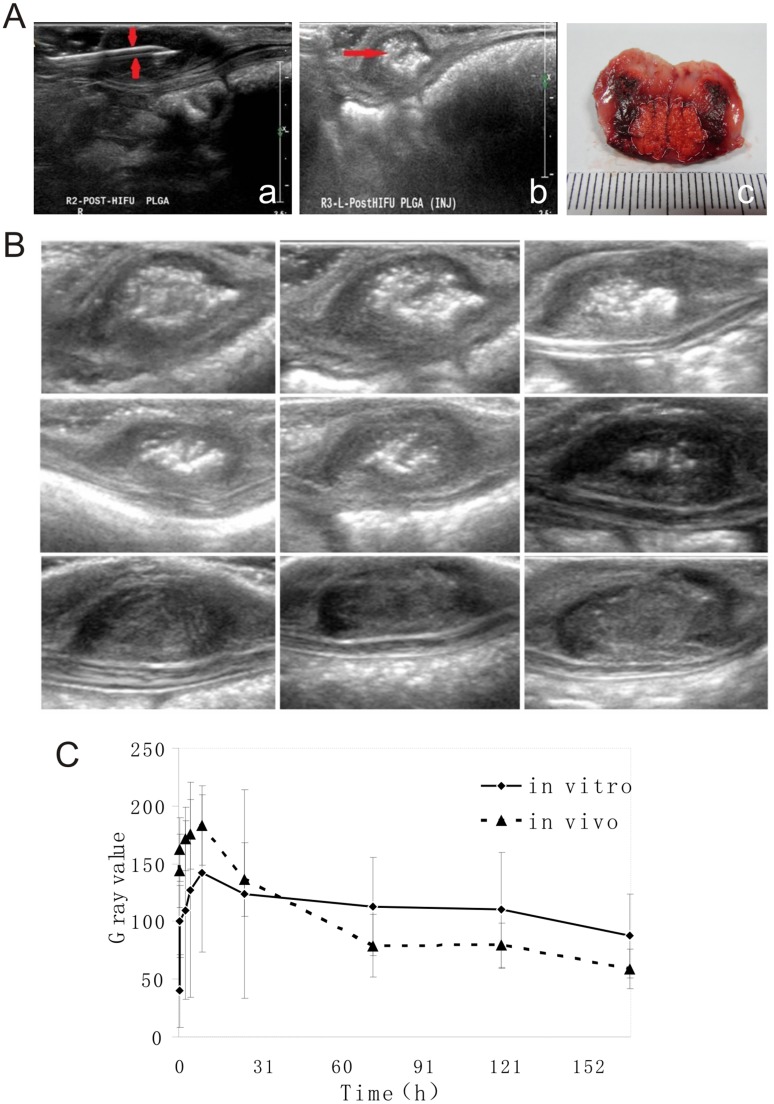
In vivo DOX-Loaded PLGA Gel. **A:** In vivo DOX-Loaded PLGA Gel Injection Imaging. A-a: B-mode ultrasound imaging of the injection process. A-b: Ultrasound imaging immediately after the injection of the DOX-loaded PLGA gel. The scale bar represents 1cm. A-c: Photograph of the cross-section of a representative tumor with injected PLGA gel; the white dashed line outlines the PLGA material in the HIFU-ablated tumor. **B**: In vivo Phase Inversion. Representative grayscale ultrasound images of the DOX-loaded PLGA gel phase inversion over time in HIFU-ablated subcutaneous VX_2_ tumors of rabbits. The scale bars represent 1cm. **C:** Grayscale Analysis of Phase Inversion. In vitro and in vivo changes in the mean grayscale value of the DOX-loaded PLGA gels over time. The data are presented as the means ± STDEV (n = 3).

The noninvasive PLGA gel injection process was made possible through the use of ultrasound imaging. Fig. 4 shows that the use of B-mode ultrasound guidance makes the needle used for PLGA gel injection clearly visible and made it possible to observe the entire process of injection (Fig. 4A-a). Fig. 4A-b indicates that the injected PLGA gel, which is also visible with B-mode ultrasound, appeared to be hyperechoic. The presence of the PLGA gel in the HIFU-ablated tumors was validated by the photographs (Fig. 4A-c).

We also evaluated the phase-inversion process of PLGA gel in vivo over time. A high echo was detected immediately after the PLGA gel was injected into the ablated tumors under two-dimensional ultrasound imaging. The contrast of the PLGA gels in the HIFU-ablated tumors waned down with time (Fig. 4B).

In both the in vitro and in vivo PLGA gel phase inversion studies, the average grayscale values of the images from each imaging time point were analyzed semiquantitatively using a Sonomath quantitative ultrasound image analyzer. Our results show that the average grayscale values of the PLGA gels in both the in vitro and in vivo studies continually increased until reaching the maximum values by 8h post injection (141.88±68.3 for in vitro and 183.13±34.5 for in vivo) and then tapered off (Fig. 4C).

### 5. In vivo tumor treatment efficacy

**5.1. Tumor volume changes after treatment**. In this section, “No Treatment” signifies that did not receive any treatment, “Sham” signifies HIFU followed by injection of drug-free PLGA gel, and “Treatment” signifies HIFU followed by injection of DOX-loaded PLGA gel. Our results show that the tumor volumes at day 0 in all three groups were comparable, and no statistically significant differences were detected among the groups. On day 4, the tumor volumes in the Sham group grew to 155% of the pretreatment volumes. Specifically, the tumors grew from 0.47±0.3 to 0.68±0.4 cm^3^ and became the largest among all three groups. During this time, the tumors from Treatment group showed the lowest growth rate of 74% (shrunk from 0.42±0.2 at day 0 to 0.30±0.2cm^3^) compared with their pretreatment volumes and were statistically significantly smaller than those from the Sham group (*p* < 0.01).

On day 8, the tumor volumes of the No Treatment group maintained their status as the largest tumors and grew to 1.42± 0.9cm^3^ (311% of their initial volumes). In contrast, the tumor volumes of the Treatment group continued to shrink to 0.26±0.1cm^3^ (77% of their initial volumes) and remained as the smallest among all three groups. Similarly, the tumor volumes of the Sham group also shrunk to 1.10±0.7cm^3^ (130% of their initial volumes), and statistically significant differences were detected between the Sham and Treatment groups (*p* < 0.05; Table 1 and Fig. 5A). The gross tumor volumes are also presented in Fig. 5A as individual photographs that more intuitively show that the tumor volumes are smallest in the Treatment group at the end of the treatment.

**Table 1:** VX_**2**_ Tumor Volumes (cm^**3**^) and Tumor Growth Rates (%).

Groups	Tumor Volume at Day 0 vs. Day 4 after Treatment			Tumor Volume at Day 0 vs. Day 8 after Treatment	
	Day 0 (cm^3^, n = 12)	Day 4 (cm^3^, n = 12)	Growth (%)	Day 8 (cm^3^, n = 6)	Growth (%)
No Treatment	0.47±0.4	0.66±0.5	155	1.42±0.9	311
Sham	0.47±0.3	0.68±0.4	155	1.10±0.7	130
Treatment	0.42±0.2^#^	0.30±0.2*	74	0.26±0.1*	77

* Statistically significantly different compared with the Sham group (*p* < 0.01 at day 4 and *p* < 0.05 at day 8). Sham: HIFU + drug-free gel, Treatment: HIFU + DOX-loaded gel.

#There were no significant differences between all three groups before the treatments.

**Fig 5:**
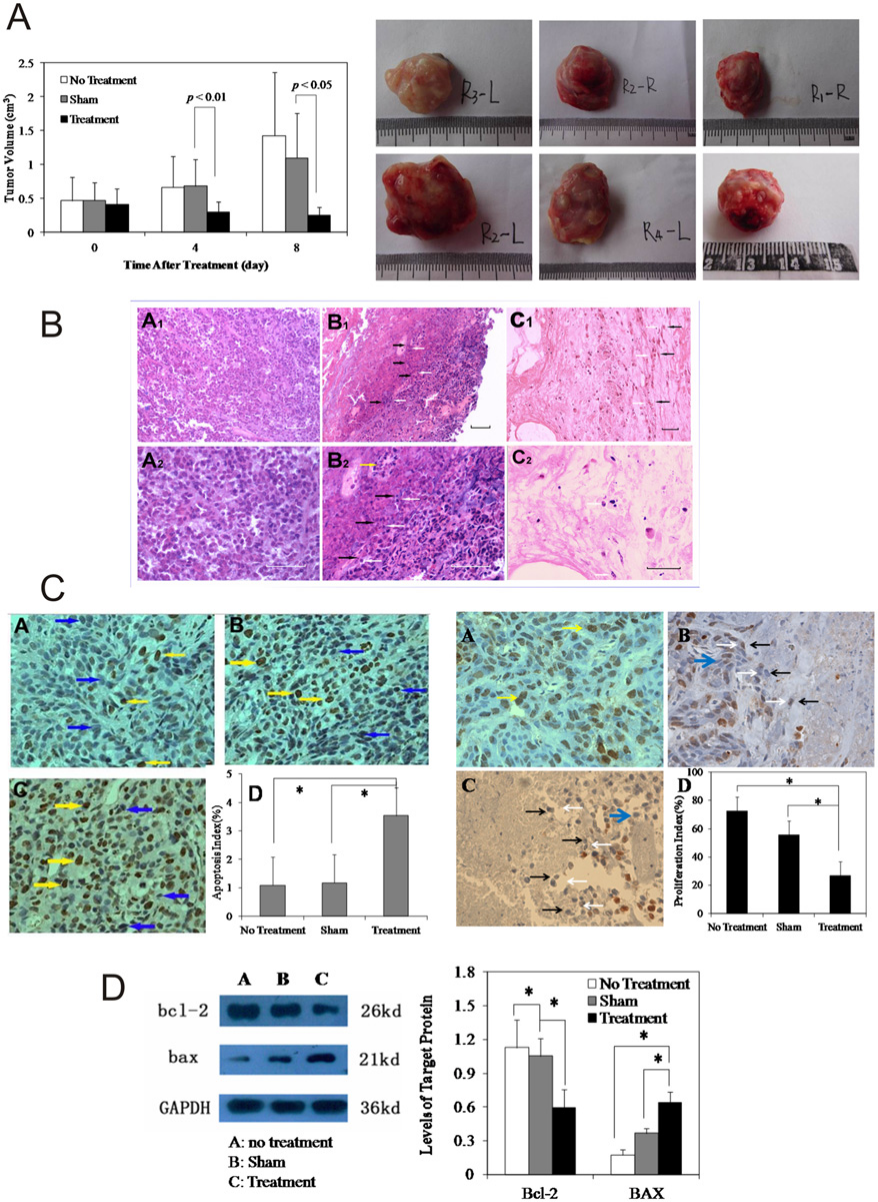
In vivo tumor treatment efficacy. **A:** Tumor Volume Changes. Sham denotes treatment with HIFU + PLGA gel without DOX. Treatment stands for HIFU + DOX-loaded PLGA gel. On days 4 and 8 after treatment, the tumor volumes of the Treatment group were statistically significantly smaller than those of the Sham group. The data are presented as the means ± STDEV. *p* < 0.05 was defined as statistically significant. **B:** H&E stained tumor tissues on Day 8 after the treatments. A: No Treatment. B: Sham. C: Treatment. The images shown in Figs. A1 and A2, B1 and B2, and C1 and C2 were acquired from the same fields of views at different magnifications: A1, B1, and C1 were acquired at 200X, and A2, B2, and C2 were acquired at 400X. The white and black arrowheads outline the interfaces of the non-ablated and the ablated tumor regions, respectively. The yellow arrow points to the tumor recurrence in the ablated tumor region. The scale bar represents 100 μm. **C**: PCNA Expression (400X magnification) and Tumor Cell Apoptosis on Day 8 after Treatment. A: No Treatment. B: Sham. C: Treatment. D: Bar graph of the proliferation index or Apoptosis Index. The scale bars represent 100μm. **D**: Western Blot of Bcl-2 and BAX Levels in Tumor Tissue 8 Days after Treatment. Left panel: Western Blots of the proteins. Right Panel: Semi-quantitative analysis of the protein levels using the grayscale values. *Statistically significant differences were detected, *p* < 0.05.

### **5.2. Histology and protein assays**. H&E stain

Tissue histology was used to provide additional insights into the effectiveness of the different treatments. Fig. 5B shows a representative image of the H&E-stained tumor tissues. A1, B1, and C1 are low-magnification overviews, and A2, B2, and C2 were acquired from the same fields of views at higher magnification. Based on the results, all three groups presented unique appearances. In the No Treatment group, the tissue architecture appeared intact, and the H&E stain was strong. The tumor cells manifested as large and irregular-shaped cells with deeply stained nuclei and were interspersed with necrotic cellular and extracellular debris. In the Sham group, there were two distinct tissue regions: HIFU-coagulated and viable tumor tissue. The boundaries of these two tissue regions are indicated by black and white arrows. In the HIFU-coagulated areas, tissue necrosis was readily visible. However, clusters of tumor cells with intact and deeply stained nuclei were also detectable. This pattern of the appearance of clusters of cells surrounded by necrotic tissues was observed in all of the samples from the Sham group. Some of these clustered cells appeared cancer-like with nuclear hyperchromatism and irregular shapes. Other cells were in the mitotic phase. In addition, multinucleated cells were also detected in these clustered cell regions. In the non-ablated tumor region, compared with tumors from the No Treatment group, there were more fractured cellular nuclei, and some apoptotic bodies were detectable. Moreover, the majority of the cells displayed the usual characteristics of VX_2_ tumors and appeared densely packed with a low grade of differentiation and a high nuclear to cytoplasmic ratio and infiltrated with cells in the mitotic phase. In the Treatment group, the tissue architecture was no longer intact. Tissue necrosis was apparent, and fibrous tissue was noted. Necrotic debris and fractured cells were visible throughout this region, and the outlines of the tumor cells were weakly visible with slight details of the cytoplasm and nuclei.

Cell proliferation and apoptosis

Cell proliferation and apoptosis were studied to further assess the treatment outcomes. Fig. 5C shows representative images. In the No Treatment group, proliferating cells, which are marked by PCNA labeling and were stained brownish yellow (chocolate or dark brown), were uniformly distributed in the two-dimensional tissue images. However, few proliferating cells were observed in the samples from the Treatment group. PCNA-positive cells were also readily detected in the Sham group, but these were mainly found in the non-ablated tumor regions. The semi-quantitative proliferation index (PI), which was calculated outside the necrotic tissue regions, of the Treatment group was significantly lower than those of the No Treatment and Sham groups (*p*<0.05). Conversely, in the Treatment group, in the regions surrounding the HIFU-coagulated tissues, there were more nuclear-cracking pyknotic cells and hyperchromatic apoptotic cells compared with the number found in the Sham and No Treatment control groups. The apoptosis index (AI: 3.53±0.3) of the Treatment group was significantly higher than those of the other two treatment groups (*p* < 0.05), as shown in Table 2 and Fig. 5C.

**Table 2:** Tumor Cell Proliferation Index (PI) and Apoptosis Index (AI) 8 days after Treatment.

Group	AI (%, n = 6)	PI (%, n = 6)
No Treatment	1.09±0.1	72.70±6.6
Sham	1.17±0.1	55.71±8.9
Treatment	3.53±0.3* ^#^	26.61±4.2* ^#^

*compared to the No Treatment group (*p* < 0.05).

# compared to the Sham group (*p* < 0.05).

The expression of Bcl-2 and Bax proteins in tumor

Cell proliferation and apoptosis were further confirmed by an analysis of the Bcl-2 and Bax protein levels using western blot methods. Our results show that the Bcl-2 levels in the Treatment group are the weakest and that those found in the No Treatment group were the strongest, although these are comparable to those of the Sham group. In contrast, the Bax levels in the Treatment group were the strongest, and those of the No Treatment group were the weakest.

After normalization to GAPDH, the Bcl-2 levels in the Treatment group were found to be significantly lower than those of the No Treatment and Sham groups (*p* < 0.05), whereas the Bax levels were significantly higher in the Treatment group compares with the other two groups (*p* < 0.05, Fig. 5D).

### 6. Local drug distribution

**Fig 6:**
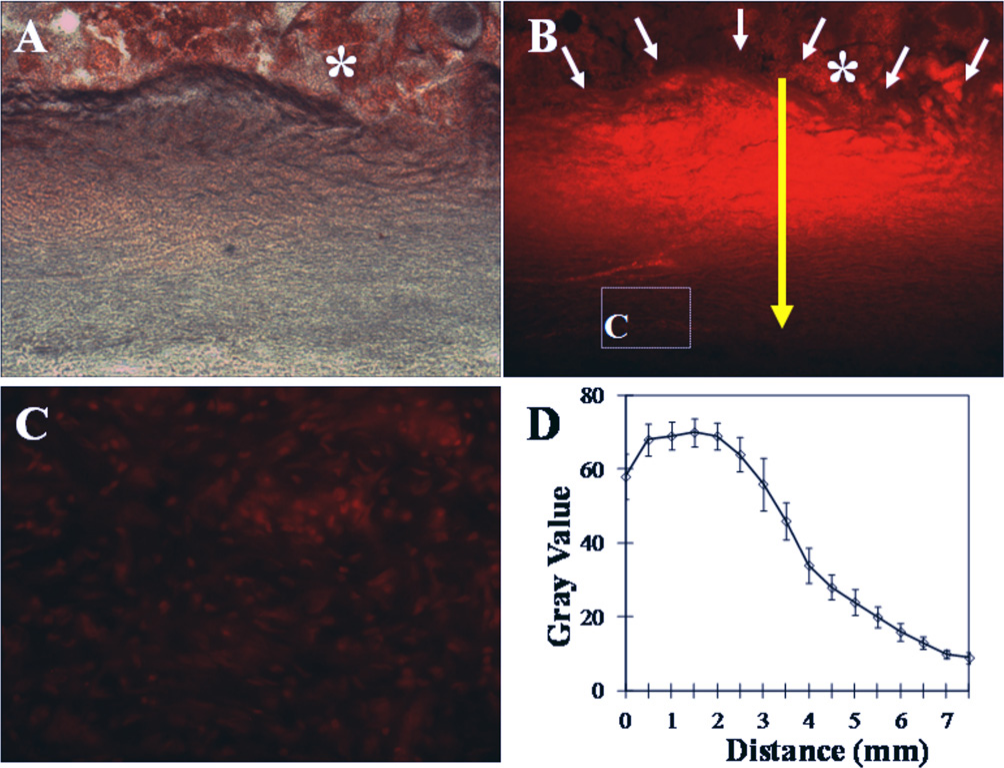
Distribution of Doxorubicin in HIFU-ablated Tumor Tissue. Zero signifies the center of the implants, and the other numerical values refer to the radial distances from the center of the implants. A: Light microscopy image of cryosectioned tumor tissue. B: Fluorescence microscopy image of the same tumor tissue section. C: High-power image of the area in the white box shown in B. D: Fluorescent intensity. The asterisk (*) indicates the PLGA implant, the white arrow indicates the edge of the PLGA implant, and the yellow arrow indicates the distance from the edge. The scale bar represents 100μm.

The imaging of cryosectioned tumors using a fluorescent microscope showed that the PLGA materials in the partially ablated tumors are readily identifiable. DOX was distributed in the ablated tumor tissues in a ladder-like manner in which the highest intensity was observed at the injection sites or the center of the PLGA implants. The DOX intensity decreased radially outward away from the implants. It is noteworthy that DOX reached cells outside the HIFU-ablated tissue region, as shown by the fluorescent signals detected in the tumor cells outside of this region. The line chart illustrates the changes in the fluorescent intensity outward from the center of the PLGA implants (Fig. 6).

## Discussion

Recent technological improvements have the rendered increased popularity of HIFU in medicine. To date, hundreds of thousands of patients from different areas, including neurosurgery, ophthalmology, urology, gynecology, and oncology, have been clinically treated with HIFU therapy with success [16–18]. The fundamental working principles of HIFU ablation are includes both thermal and mechanical means. This methodology thermally coagulates the tissue of interest via the absorption of the ultrasound energy during transmission and mechanically destructs the target tissue via cavitation effects. HIFU has been promoted as a completely noninvasive procedure due to its extracorporeal nature. Due to its features, such as little pain involvement, low cost, short recovery period, no scar tissue formation, and no dosing limitation, HIFU has been gaining momentum as an alternative for the treatment of primary solid tumors and metastatic diseases. Although an increasing number of research efforts have reported promising results, it is becoming evident that the technique needs some refinement, particularly in focal heat ablation applications, due to the incomplete eradication of tumor tissue.

In this work, we explored the idea of using injectable, in situ-forming, and phase-inverting DOX-loaded PLGA implants to treat residual tumors after HIFU ablation and prevent tumor recurrence. To establish this work, extensive efforts were first placed into the formulation and characterization of the PLGA drug delivery systems in both in vitro and in vivo settings. We then optimized the in vivo HIFU ablation parameters using contrast-enhanced ultrasound imaging before conducting in vivo efficacy studies in which HIFU ablation combined drug-free PLGA was compared to HIFU ablation combined with DOX-loaded PLGA and no treatment controls.

During the initial phase of the project, our efforts were devoted to the determination of the PLGA formulation. Three different combinations of NMP/PLGA/DOX at mass ratios of 80 (or 60 or 40)/15 (or 35 or 55)/5 were formulated. However, the preliminary results from in vivo studies provided two key findings. First, the formulation with the lowest mass ratio of PLGA caused the death of rabbits due to the formation of vascular thrombosis (data not shown). Second, the injectability of the PLGA formulation was negatively correlated to the PLGA mass ratio, i.e., a higher PLGA content made the injection more difficult but resulted in a lower chance for vascular thrombosis formation. Hence, all of the results presented in this manuscript were acquired using the formulation with 55% (wt) PLGA.

After the PLGA formulation was selected, we shifted our efforts to the characterization of the drug release and phase-inversion processes of the drug delivery system. Although the concept of using a controlled and sustained drug delivery system is straightforward, the design of such systems often proves challenging because drug release processes by these delivery systems are governed by the interactions of a variety of factors, including solvent [19], solute [20], excipient [21], and local environments [22]. In addition, most characterization studies of such systems are conducted in vitro, and behaviors extrapolated from such artificial environments are often poorly translated into in vivo settings. In this study, we employed the most current knowledge regarding PLGA drug delivery systems and implemented it into our design. Studies have shown that drug release from in sit*u*-forming PLGA systems are polymer precipitation rate-dependent, and a recent study presented a novel means using diagnostic ultrasound to noninvasively and nondestructively monitor PLGA precipitation processes [23]. By employing this technique, we evaluated the phase-inversion processes of the proposed drug delivery system. The results showed that the in vitro and in vivo characteristics of the PLGA gel phase inversion are comparable. The average grayscale values of the PLGA gels continually increased until reaching their maximum value by 8h (141.88±68.3 for in vitro and 183.13±34.5 for in vivo) and then started to taper. These findings are in agreement with data reported by a previous report that showed that the in vitro and in vivo fluorescein release profiles from PLGA implants are comparable at least during the first 8h. The authors suggested that the drug release is governed by polymer precipitation during this time. In the same study, the researchers presented data showing that the in vivo drug release after 8h was significantly faster than that observed in vitro and speculated that the discrepancy may be caused by differences in the surface areas and convective removal of the solvent and drug. Our in vitro DOX release studies showed that 87.99±4.3% of the loaded DOX was released by day 4. Based on the results reported by Solorio et al., the majority of DOX may have been released sooner in vivo, suggesting that our use of day 8 as the therapeutic endpoint in our work is adequate.

We conducted in vivo efficacy studies in a rabbit subcutaneous tumor model using hepatic VX_2_ tumors. Before undertaking our in vivo efficacy work, the optimal parameters for in vivo HIFU ablation were determined to induce substantial tissue coagulation necrosis but create an incomplete treatment scenario in order to represent the clinical issue of tumor recurrence. To maintain the noninvasive nature of HIFU ablation, diagnostic ultrasound was used to guide the needles for PLGA injection in vivo in real time, and the feasibility of this type of undertaking was determined. The use of B-mode ultrasound guidance made the needle used for the PLGA gel injection clearly visible and allowed the observation of the entire process of injection in real time.

Contrast-enhanced ultrasound was used to assess the treatment effects of HIFU therapy and to help determine the optimal treatment parameters for HIFU ablation. Our results show that VX_2_ tumors without HIFU ablation and without contrast enhancement are uniformly clump-like and hypoechoic. These tumors appeared elliptical in shape with clear tumor and normal tissue boundaries. However, HIFU ablation increased the tissue echo, and the initially hypoechoic tissue became hyperechoic. In addition, without HIFU ablation, the microbubble contrast agents uniformly penetrated the entire tumor volume and enhanced tissue contrast within 5–10s after administration, suggesting that the tumor tissue was viable. This observation was validated by TTC staining of the tumor sections. In contrast, after incomplete ablation, the contrast enhancement in the residual tumors tissues was irregular and absent in the HIFU-ablated region. Again, the TTC method was used to confirm this interpretation. In this later case, TTC failed to stain the HIFU-ablated tissue region, whereas the non-ablated tissue region appeared rosy red in color.

To gain insights into the treatment efficacy, the tumor volume changes were monitored using diagnostic ultrasound. Our results show that both adjunct treatment (Treatment group) and sham treatment (Sham group) improved the tumor response compared with no treatment (No Treatment group). However, the tumors treated with HIFU and DOX-loaded PLGA exhibited a considerably greater reduction in lesion size than the sham-treated control tumors.

To gain additional insights into the effectiveness of the different treatments, ex vivo tissue histology was performed. The H&E-stained tissue sections showed that the tissue architecture after treatment with the combination of HIFU and DOX-loaded PLGA was no longer intact. Tissue necrosis was apparent, and fibrous tissue was detectable. Necrotic debris and fractured cells were visible throughout the tissue sample, and the outlines of tumor cells were weakly visible with slight details of the cytoplasm and nuclei, suggesting that the treatment is effective. Interestingly, in the Sham control group, two distinct tissue regions were observed: one was HIFU-coagulated, and the other was viable non-ablated tumor tissue. In the HIFU-coagulated areas, tissue necrosis was readily visible. However, clusters of cells with intact and deeply stained nuclei surrounded by necrotic tissues were also detectable in all tissue samples belonging to the Sham group. The majority of these cells displayed the usual characteristics of VX_2_ tumors, i.e., they appeared densely packed with irregular shapes and with a low grade of differentiation, a high nuclear to cytoplasmic ratio, and infiltration with cells in the mitotic phase. In addition, multinucleated cells were also detectable in these clustered cell regions. All of these cell and tissue characteristics were also readily detectable in the tissues belonging to the No Treatment group, suggesting the high rate of tumor recurrence in the Sham control group. In contrast, no such observations were made for the Treatment group, which further supports that the treatment resulted in a better treatment outcome.

PCNA and TUNEL assays were used to obtain information on cell proliferation and apoptosis and hence to further assess the outcomes of the treatments. Under exactly the same HIFU ablation conditions, there were more nuclear cracking pyknotic cells and hyperchromatic apoptotic cells in the samples from the Treatment group, particularly in the regions surrounding the HIFU-coagulated tissue. In addition, a small number of proliferating cells were observed in these regions. However, there were less apoptotic but more proliferating cells in these tissue regions after sham treatment. Using western blot methods, we validated these findings and showed that the level of Bcl-2, an anti-apoptotic protein and oncogene, was the lowest, and the level of BAX protein, the apoptosis promoter, was the highest in the tumor tissues treated with HIFU ablation combined with DOX-loaded PLGA compared with the other two groups. Studies by Shan Ke et al. have shown that low-grade temperature at the target sites can facilitate the rapid progression of residual hepatic VX_2_ tumors [24]. Although this work may help explain why there are more proliferating cells in the Sham group, it also demonstrates the critical role of DOX-loaded PLGA gels in the eradication of residual tumors. Our results show that DOX is distributed in ablated tumor tissue in a ladder-like manner in which the highest concentration of DOX are detected at the injection sites and the concentration then tapered off in the outward direction. Our results also show that the tumor cells outside the ablated regions are lighted up under a fluorescent microscope, suggesting the DOX is able to diffuse away from the implants and reach cells outside the ablated regions.

Both in vivo and ex vivo results have shown that HIFU followed by in situ-forming DOX-loaded PLGA produce significantly more desirable outcomes and prevent tumor recurrence. The enhanced response of tumors following the combined treatment indicates that the design of the specific mass ratio (40/55/5) of the NMP/PLGA/DOX formulation was a success. In summary, this work provided valuable insights into the application of in situ-forming drug-loaded PLGA for the treatment of potential but detrimental residual tumors after HIFU ablation and the prevention of tumor recurrence. However, the authors are aware of the limitations of the present study. Using a subcutaneous tumor model in rabbits is straightforward, reproducible, and convenient for conducting controlled scientific experiments, but the subcutaneous location does not account for the typical physiological processes affecting the focal heat ablation treatment. In addition, a long-term survival study instead of an 8-day observation period will provide more convincing evidence of the true nature of the combined treatment regimen. Finally, a temperature mapping may help draw more convincing conclusions under specific temperature conditions. Nonetheless, the findings from the present study reveal a novel second line of defense for the prevention of tumor recurrence after HIFU ablation that is worthy of pursuit and hence further investigation.

## Conclusion

This paper describes a novel adjunct regimen for the treatment of solid VX_2_ tumors that uses doxorubicin-loaded, injectable, in situ-forming, and phase-inverting PLGA implants as the second line of defense to prevent tumor recurrence after HIFU. Our results show that the proposed adjunct treatment regimen leads to a more significant shrinkage of tumor volumes compared with the sham control treatment, even though partial tumor ablation was intentionally produced to assess the treatment effects. More compelling results from tissue histology and western blot assays provided complimentary evidence that further support the hypothesis that the adjunct regimen is significantly more effective than HIFU treatment alone. However, our study is not without limitations, and the treatment efficacy of this regimen in a longer-term survival study should provide more definitive answers on whether this adjunct regimen is worthy of further investigation. In addition, a more physiological relevant tumor location, such as the liver instead of a subcutaneous location, will provide better insights into the behavior of the PLGA implants. Such studies are underway in our laboratory and will be presented in another manuscript. Nevertheless, to the best of our knowledge, this study provides the first demonstration of the potential of the combination of HIFU with an in situ-forming PLGA implant with compelling positive in vivo efficacy data.
